# Molecular Mechanism of Ischaemic Preconditioning of Skeletal Muscle In Vitro

**DOI:** 10.7759/cureus.3763

**Published:** 2018-12-21

**Authors:** Ciara Fox, Pauline Walsh, Kevin J Mulhall

**Affiliations:** 1 Orthopaedics, Royal College of Surgeons in Ireland, Dublin, IRL; 2 Orthopaedics, Orthopaedic Research and Innovation Foundation, Dublin, IRL

**Keywords:** ischaemic preconditioning, ischaemia-reperfusion injury, skeletal muscle, hypoxic chamber

## Abstract

Introduction

Ischaemic preconditioning (IPC) is a phenomenon whereby tissues develop an increased tolerance to ischaemia and subsequent reperfusion if first subjected to sublethal periods of ischaemia. Despite extensive investigation of IPC, the molecular mechanism remains largely unknown. Our aim was to show genetic changes that occur in skeletal muscle cells in response to IPC.

Methods

We established an in vitro model of IPC using a human skeletal muscle cell line. Gene expression of both control and preconditioned cells at various time points was determined. The genes examined were hypoxia-inducible factor-1 alpha (HIF-1 alpha), early growth response 1 (EGR1), JUN, and FOS. HIF-1 alpha is a marker of hypoxia. EGR1, JUN, and FOS are early response genes and may play a role in the protective responses induced by IPC.

Results

HIF-1 alpha was upregulated following one and two hours of simulated ischaemia (p = 0.076 and 0.841, respectively) verifying that hypoxic conditions were met using our model. Expression of EGR1 and FOS was upregulated and peaked after one hour of hypoxia (p = 0.001 and <0.00, respectively). cFOS was upregulated at two and three hours of hypoxia. IPC prior to simulated hypoxia resulted in a greater level of upregulation of EGR1, JUN and FOS genes (p = <0.00, 0.047, and <0.00 respectively).

Conclusion

This study has supported the use of our hypoxic model for studying IPC in vitro. IPC results in a greater upregulation of protective genes in skeletal muscle cells exposed to hypoxia than in control cells. We have demonstrated hitherto unknown molecular mechanisms of IPC in cell culture.

## Introduction

Ischaemic preconditioning (IPC) is a phenomenon whereby tissues develop an increased tolerance to ischaemia and subsequent reperfusion if first subjected to sublethal periods of ischaemia. The concept of IPC was first introduced in relation to cardiac tissue by Murry et al. in 1986 [1]. Canine hearts were subjected to four cycles of five minutes of ischaemia followed by five minutes of reperfusion as a means of preconditioning. This was then followed by a further sustained 40-minute period of ischaemia. Those hearts that were subjected to “ischaemic preconditioning” were found to have a statistically significant 70% smaller infarct size when compared to controls.

Following Murry’s pivotal paper, there have been extensive investigations into the use of IPC, primarily in the cardiac setting. IPC was established as a powerful and reproducible mechanism of providing cardioprotection, however, its clinical use was limited due to the invasive nature of preconditioning the myocardium. The concept of “remote” ischaemic preconditioning was an approach which has the potential to be non-invasive. The validation that one vascular bed could precondition another distant or “remote” vascular bed originated from a study by Przyklenk et al. in 1993 [2]. They proved that preconditioning the vascular bed supplied by the circumflex artery in canines reduced the infarct size in the left anterior descending (LAD) vascular bed following sustained LAD occlusion. Therefore, a tissue subjected to brief episodes of ischaemia/reperfusion results in a greater tolerance to sustained ischaemia not only by that same tissue but also tissues distant or “remote” to it. This has allowed for the application of IPC in a much less invasive manner. For instance, transient limb ischaemia has been shown to confer remote preconditioning to the heart [3]. In the setting of orthopaedic surgery, Addison et al. showed that skeletal muscle can be globally protected following a preconditioning stimulus applied to a limb via simple tourniquet use [4]. The protection produced by IPC can now be applied to a vast array of tissues without any invasive procedures.

Further studies have found that ischaemic preconditioning occurs in two phases – early and late protection. In 1993, Marber et al. showed that the acute phase of protection (one-two hours) was followed by a second window of protection at 24 hours [5]. This second window of protection, lasting approximately 72 hours, was shown to be clinically significant [6]. This was referred to as “delayed” or “late” preconditioning. The early phase of protection conferred by IPC is termed “classic preconditioning” [7].

Clinical application

While there is a significant body of research to support the benefit of IPC in the setting of cardiothoracic surgery [1, 8, 9], it has also been shown to be of benefit in several other tissues – including human liver [10], lung [11] and brain [12]. In addition, it plays a significant role in skeletal muscle [13, 14]. IPC improves overall contractility, force, performance and fatigue of muscle as well as protecting against ischaemia/reperfusion injury [15]. It has meaningful clinical potential in orthopaedic surgery where many operations performed on limbs are done so under tourniquet control producing ischaemic conditions and subjecting limbs to ischaemia-reperfusion injury post-op. Reducing ischaemic insult to skeletal muscle via IPC may have significant clinical benefits for orthopaedic patients.

Molecular mechanism

Despite extensive research into IPC since the 1980s, the mechanism involved in IPC protection remains poorly understood. It is thought that certain triggers, released as a result of the preconditioning stimulus, provoke a systemic response and activate a cascade of intracellular mediators and signaling molecules. These mediators produce the end-point of limiting reperfusion apoptosis and reducing ischaemic necrosis.

There are multiple chemical agents which are all hypothesized to play a role in the protective mechanism of IPC. These include adenosine, acetylcholine, angiotensin II, nitric oxide, free radicals, bradykinin, catecholamines, endothelin, opioids, and heat shock proteins [16]. It is thought that these act via signaling pathways and reduce tissue injury by reducing energy consumption, oxidative stress and apoptosis.

IPC modifies gene expression both immediately (early phase) and at delayed time points. Konstantinov et al. revealed suppression of genes coding for proteins involved in apoptosis, cytokine synthesis, leukocyte chemotaxis, adhesion and migration at 15 minutes following a remote preconditioning stimulus and further suppression after 24 hours [17]. This corresponds to the effects of both immediate and delayed preconditioning. Genes involved in protection against oxidative stress are up-regulated while pro-inflammatory genes are suppressed following IPC and subsequent ischaemia [17].

IPC results in activation of protective signaling pathways and reduction of oxidative stress, cell death and inflammation. No single factor is responsible for the protection conferred by IPC. The effects of IPC are the result of interplay between several triggers, mediators and protective genes. The cumulative action of these is a systemic response in the body which reduces injury following insult.

Objectives

Our group have previously established the use of an in vitro model of IPC [18] allowing the mechanism of IPC to be examined at a purely cellular level. We have also identified several genes that are upregulated following IPC in total knee arthroplasty patients (available to us on microarray) [19]. Our aim was to further examine the molecular mechanisms involved in IPC of skeletal muscle in vitro, using our hypoxic chamber model. We focused on the role of early response genes – early growth response-1 gene (EGR1), CJUN (the protein coded for by JUN gene), and CFOS (a proto-oncogene). A greater understanding of the molecular basis of IPC will allow for future therapies to be used which target this effect.

## Materials and methods

Human skeletal muscle cell line and culture

Normal human skeletal muscle cells were derived from the pectoralis major muscle of a single Caucasian male donor. The cells were purchased from Promocell (www.promocell.com) as a cryopreserved batch of one million cells and subcultured to passage 4 (P4) and passage 5 (P5). This built up a stock of P4 and P5 cells from which all experiments were conducted. They were maintained as adherent cultures in 18mls of skeletal muscle growth medium (also supplied by Promocell) at 37°C in a humidified incubator containing 5% CO_2_. Culture media was replaced every two days. Cells were seeded at a density of 1 x 10^5^/well in six-well plates. All experiments were carried out 48 hours after seeding and were performed in triplicate.

Three groups were assigned; an absolute control, an ischaemic control and a preconditioned group. All steps were performed under aseptic conditions in a laminar flow cabinet.

Hypoxia chamber

**Figure 1:**
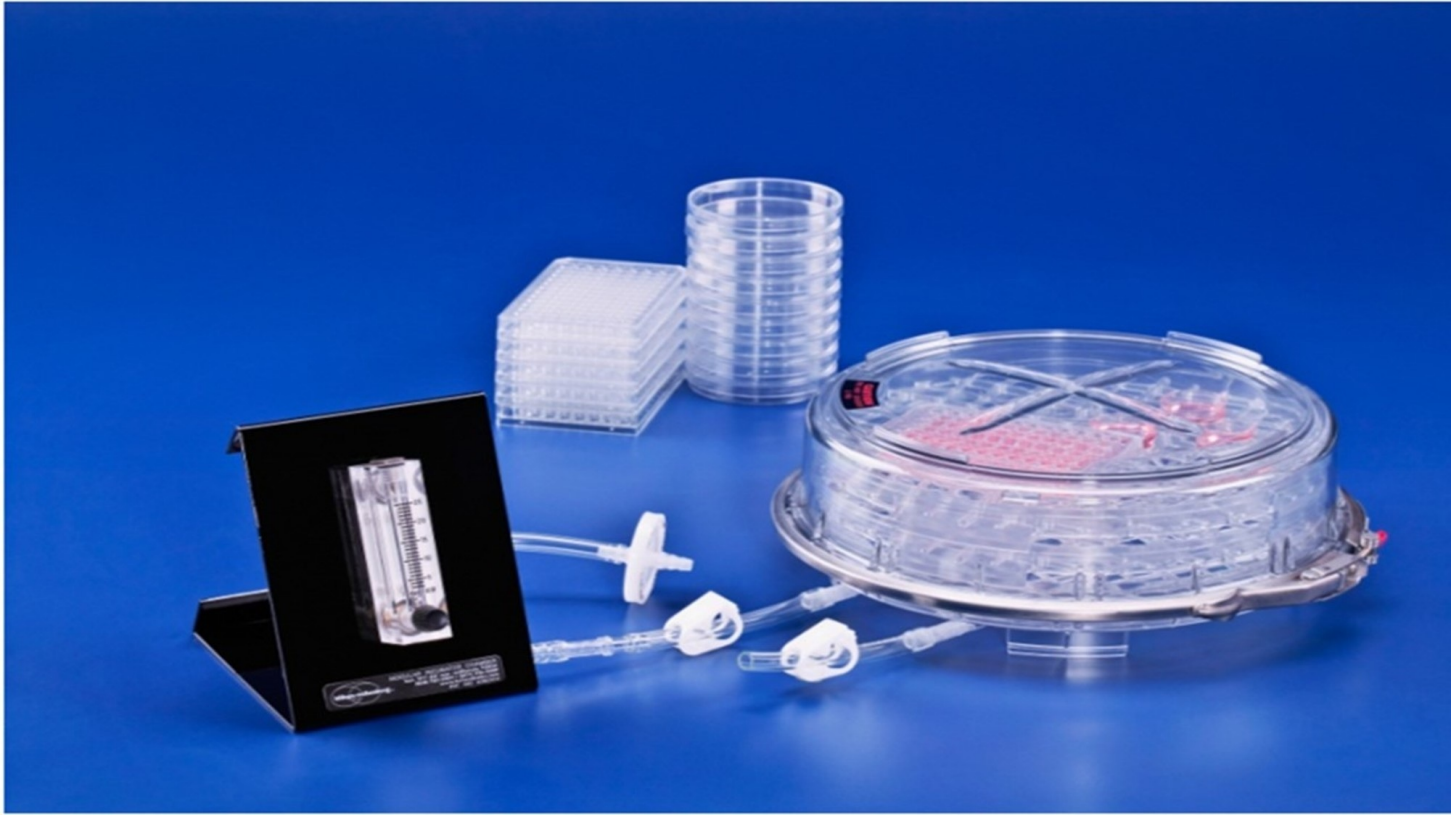
Hypoxic chamber and flow meter.

The chamber used to maintain the established hypoxic environment is an eight litre, air-tight re-sealable chamber with in-built ergonomic features to allow quick intra-chamber gas exchange. It is manufactured by Billups-Rothenberg Inc. (San Diego, CA, USA) (www.brincubator.com). A flow meter was connected between the gas source and the chamber to quantify the current of gas input to the chamber. The flow meter was supplied along with the chamber by the company (Figure 1).

**Figure 2:**
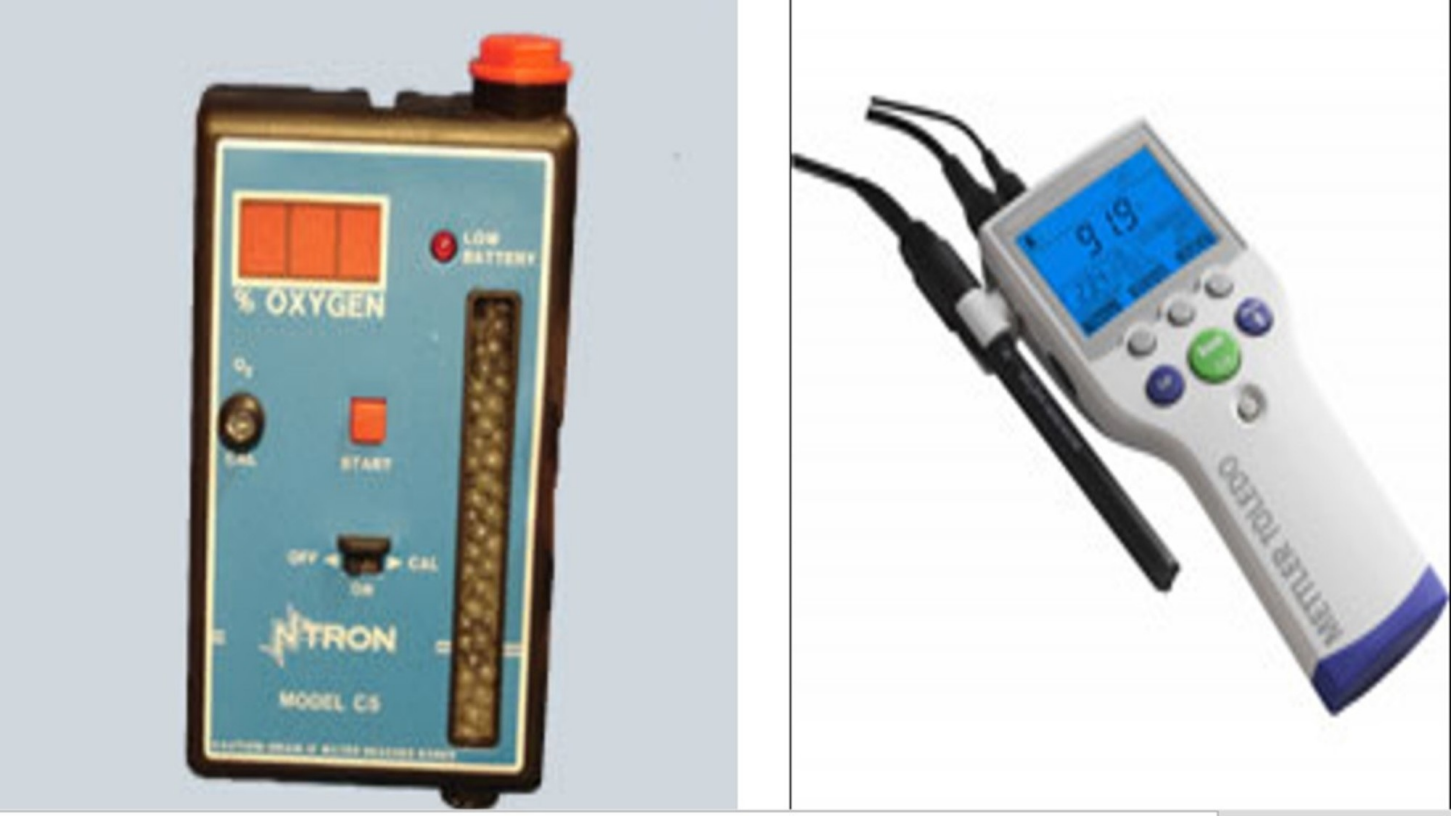
Ambient oxygen sensor and dissolved oxygen sensor.

The use of this chamber to study IPC in vitro has previously been established by our research group [18] and has been utilised by others [20] to produce dependable ischaemia. A hypoxic media was produced by bubbling 1% oxygen bubbled through Dulbecco’s buffer solution (Sigma-D8662). The decision to use 1% oxygen is based on research by Wilgis [21]. Hypoxic conditions were then verified using two oxygen meters, one to assess oxygen content within the ambient gaseous surroundings and the other to assess oxygen content dissolved within a liquid (Figure 2).

Experimental protocol

**Figure 3:**
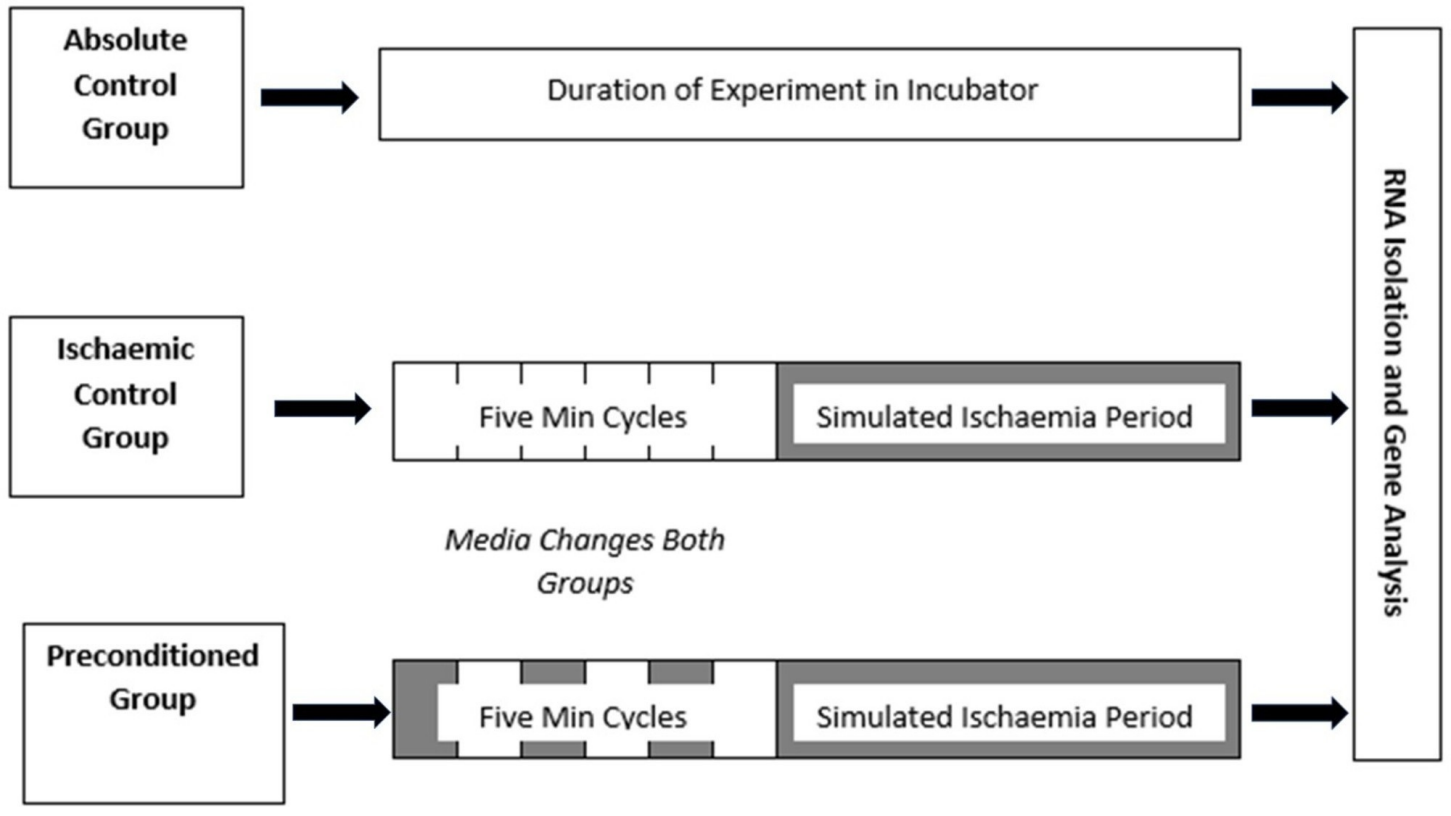
Outline of in vitro experiment.

The preconditioning protocol consisted of three cycles of five minutes ischaemia/five minutes reperfusion (Figure 3). The decision to use three cycles of five-minute periods was based on previous studies which used the same protocol to examine IPC in skeletal muscle [13, 22]. The simulated ischaemia environment was created by exchanging the cell growth media for warm de-oxygenated Dulbecco’s phosphate buffered saline solution and placing the plate in the hypoxia chamber. The chamber was flushed for five minutes then sealed tight and placed in the 37°C incubator for five minutes to keep the cells warm. The simulated reperfusion environment was created by removing the cells from the hypoxia chamber, exchanging the buffer for warm normoxic skeletal growth medium and placing back into the incubator for five minutes. The ischaemic control group was exposed to changes of normal growth media at the same time as the ‘preconditioned group’ was receiving media-buffer exchanges. This controlled for any mechanical preconditioning effect produced by media exchanges. At the end of the preconditioning cycles both the ‘preconditioned group’ and ‘ischaemic group’ were exposed to a simulated ischaemic environment for a variable length of time. This was spent in the 37°C humidified incubator.

Choice of genes

Induction of Hypoxia

The first component of assessing IPC in vitro using a human skeletal muscle cell line required verifying that the hypoxic chamber being used was producing adequate hypoxic conditions (Figure 1). During our experiments, both the ambient oxygen and the dissolved oxygen sensors confirmed that 1% oxygen levels were present within the hypoxic chamber and in the buffer solution (Figure 2).

Firstly, we verified induction of hypoxia via hypoxia-inducible factor-1α (HIF-1α), a transcription factor regulating response to hypoxia. In hypoxia, HIF-1α degradation is inhibited resulting in an upregulation of HIF-1α expression [23]. It acts in a similar manner to early response genes in that it is upregulated early when cells are in hypoxic conditions but it is only transiently upregulated [24]. In addition to increased protein expression, HIF-1α is upregulated on a messenger RNA (mRNA) level in response to hypoxia both in vitro and in vivo [25]. We measured the expression of HIF-1α mRNA in order to verify that our experimental procedure was indeed producing cellular hypoxia. Comparison of HIF-1 α expression in hypoxic samples was made to that of control samples and results plotted as fold change. GAPDH (Glyceraldehyde 3-phosphate dehydrogenase) was used as a relative marker for both control and hypoxic groups as the “housekeeping gene”.

Early Response Genes to Compare Effect of Hypoxia Alone to IPC + Hypoxia

Work by our group previously identified several early response genes as being upregulated following an ischaemic preconditioning stimulus in patients undergoing primary total knee arthroplasty [19]. In this study, we specifically targeted EGR-1, CFOS, and CJUN to compare gene expression in vitro following simulated hypoxia alone to that of IPC and hypoxia. These early response genes are upregulated rapidly and have previously been shown to play a role in the protective response to ischaemic preconditioning. EGR-1, CFOS, and CJUN are all involved in the cellular adaptation to ischaemia and have been shown to protect cells from ischaemia in cardiac models [26]. However, the prolonged induction of these genes may have deleterious consequences and has been linked to induction of apoptosis [27]. The early upregulation and subsequent downregulation in expression of these genes may be an important element in IPC.

Gene expression of an absolute control, an ischaemic control and a preconditioned group of cells at various time points was determined. The expression of these genes was compared to previous micro-array data [19] to ensure that the genomic response of skeletal muscle cells to ischaemia was reproducible in vitro.

Gene expression analysis

RNA was extracted from cell samples using Tri-reagent purchased from Sigma (sigmaaldrich.com). Removal of contaminating DNA from RNA samples was performed using the DNA-free TM kit purchased from Applied Biosystems (Forster City, CA, USA) (Cat# AM1906). The generation of c-DNA (complementary DNA) from RNA was performed using a reverse transcription kit purchased from Sigma (St. Louis, MO, USA). This included dNTP (deoxyribonucleoside triphosphate - D7295), oligo dT (oligo deoxythymine - O4387), 10X buffer (B1175) and the enzyme Avian reverse transcriptase (A4464). Finally, real-time polymerase chain reactions (PCR) were performed with gene-specific markers and the Quantitect SYBER green PCR Master Mix (Qiagen UK). Oligonucleotides were designed based on sequences obtained from the Quantitative PCR Primer Database (QPPD) (Table 1). This web-based programme provides information about primers and probes that can be used to quantitate human mRNA by RT–PCR. Protein extraction was performed using CellLytic M mammalian cell lysis/extraction reagent supplied by Sigma.

**Table 1:** Forward and reverse primers used for real-time polymerase chain reactions (PCR). F = Forward primer; R = Reverse primer; EGR1 = Early growth response 1 gene; GAPDH = Glyceraldehyde 3-phosphate dehydrogenase.

Gene	Primer Sequence
EGR-1	F: 5’-AGCCCTACGAGCACCTGAC-3’ R: 5’-AGCGGCCAGTATAGGTGATG-3’
CJUN	F: 5’-GAGCGGACCTTATGGCTACA-3’ R: 5’-TGAGGAGGTCCGAGTTCTTG-3’
CFOS	F: 5’-CAAGCGGAGACAGACCAAC-3’ R: 5’-GAGCTGCCAGGATGAACTC-3’
GAPDH	F: 5’-GAGTCAACGGATTTGGTCGT-3’ R: 5’-TTGATTTTGGAGGGATCTCG-3’

**Figure 4:**
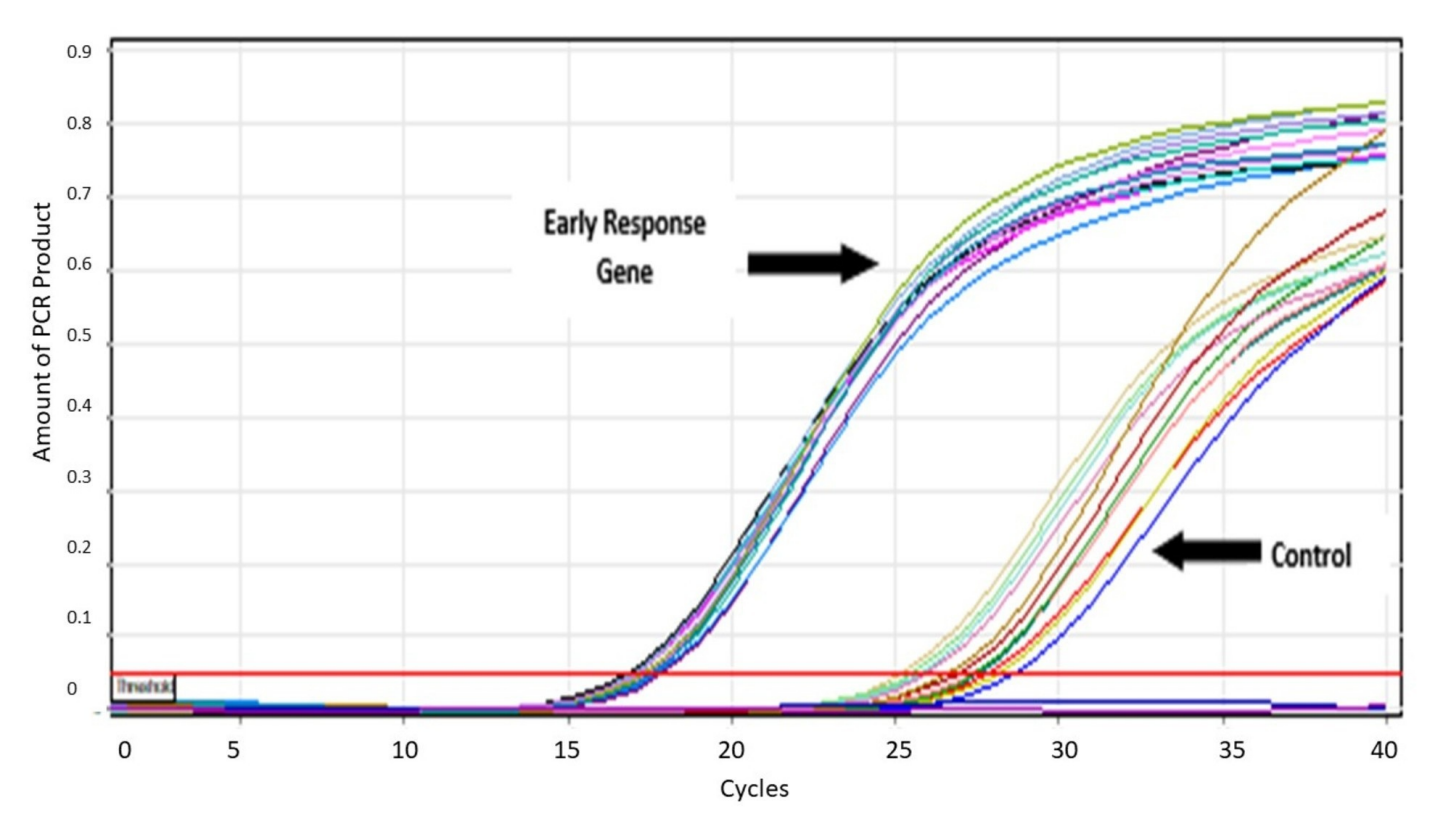
A representative polymerase chain reaction (PCR) run using complementary DNA (cDNA) of skeletal muscle cells following one hour of simulated ischaemia. The genes of interest cross the threshold earlier indicating that they are upregulated in comparison to control specimens. The close proximity of all the gene samples to each other and all of the control samples to each other indicates accuracy.

The mRNA expression was reported as a function of crossing threshold (Ct), the cycle number at which PCR amplification becomes linear. When upregulated, genes should have a lower crossing threshold on real-time PCR when compared to controls (Figure 4).

Expression of early response genes following simulated ischaemia +/- preceding IPC were compared to control samples and results plotted as fold change relative to controls. GAPDH was used as the “housekeeping gene” for both control and ischaemic groups. GAPDH was used as the “housekeeping gene” for gene expression comparison as it is a reliable reference gene for real-time PCR in several different experimental conditions, including hypoxia [28].

Statistical analysis

All data was compiled in Excel. SPSS analytical software was used for statistical analysis. The two-sample t-test was used to check for statistically significant differences in expression of genes compared to control samples. This could only validly be used when observations in each group were normally distributed and the variances were equal. In cases where the data was non-normal or if the variances were unequal, the Mann-Whitney U-test and Welch’s t-test were used respectively. Differences were considered significant if p < 0.05.

## Results

Verification of hypoxia

**Figure 5:**
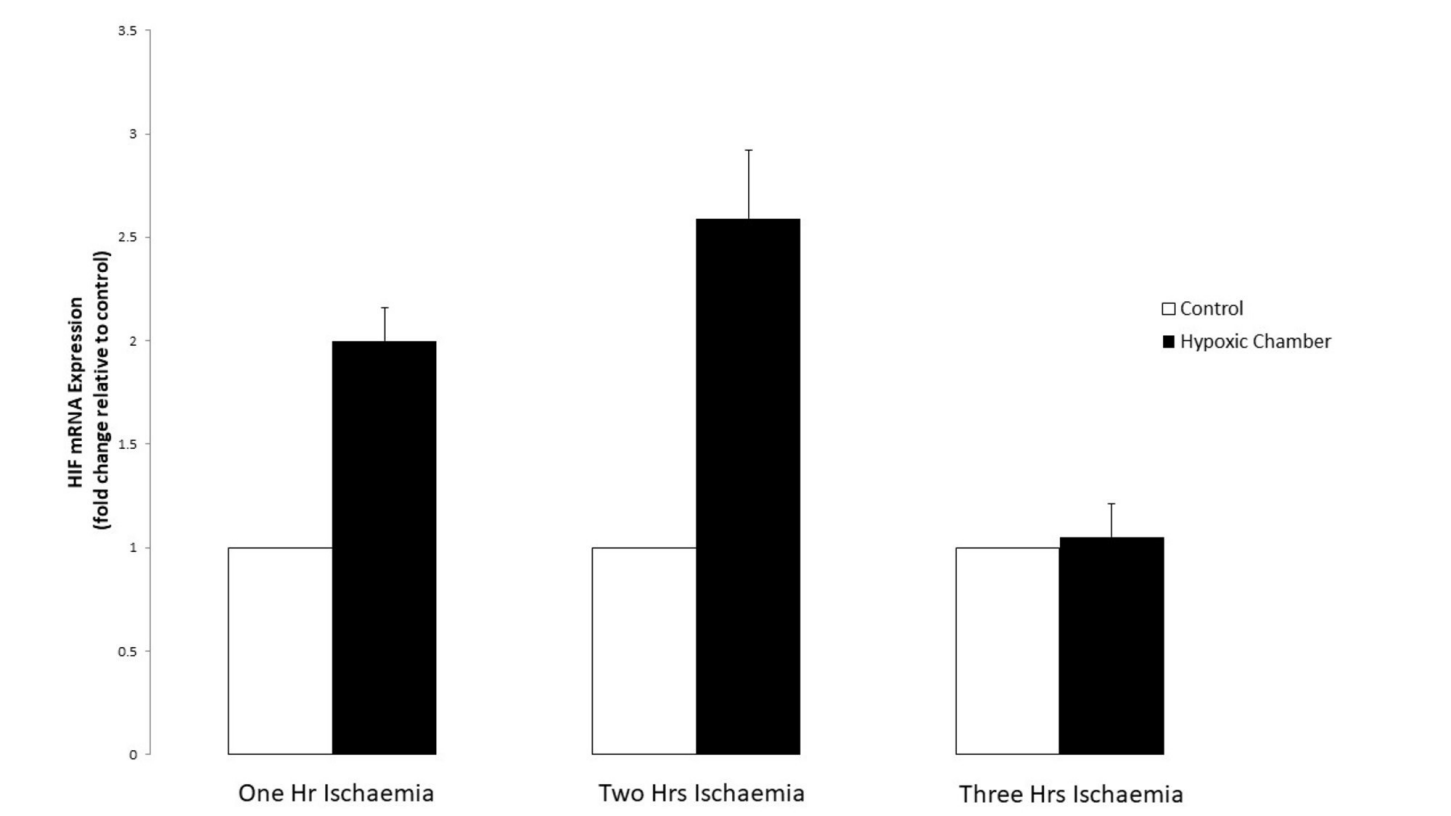
Effect of simulated ischaemia on HIF1-alpha mRNA expression. Analysis of hypoxia inducible factor-1 alpha (HIF-1α) mRNA expression was performed using real-time PCR. Gene expression is presented as a fold-change +/- Standard Deviation relative to control and represents the results of two independent experiments each carried out with triplicate samples. Means were compared using Welch’s t-test.

To examine whether our in vitro experimental conditions were reliably simulating a hypoxic environment for the skeletal muscle cells, the expression of HIF-1 α was assessed following one, two and three hours of simulated ischaemia. Analysis of HIF-1 α mRNA expression revealed a two-fold upregulation following one hour of simulated ischaemia when compared to control cells (p = 0.076). Following two hours of simulated ischaemia, HIF-1 α mRNA expression was further upregulated by 2.59 compared to controls (p = 0.841). However, HIF-1 α mRNA expression following three hours of simulated ischaemia was similar to that of control cells at 1.05 (p = 0.875) (Figure 5).

Hypoxia alone versus IPC for early response genes

Hypoxia Alone

**Figure 6:**
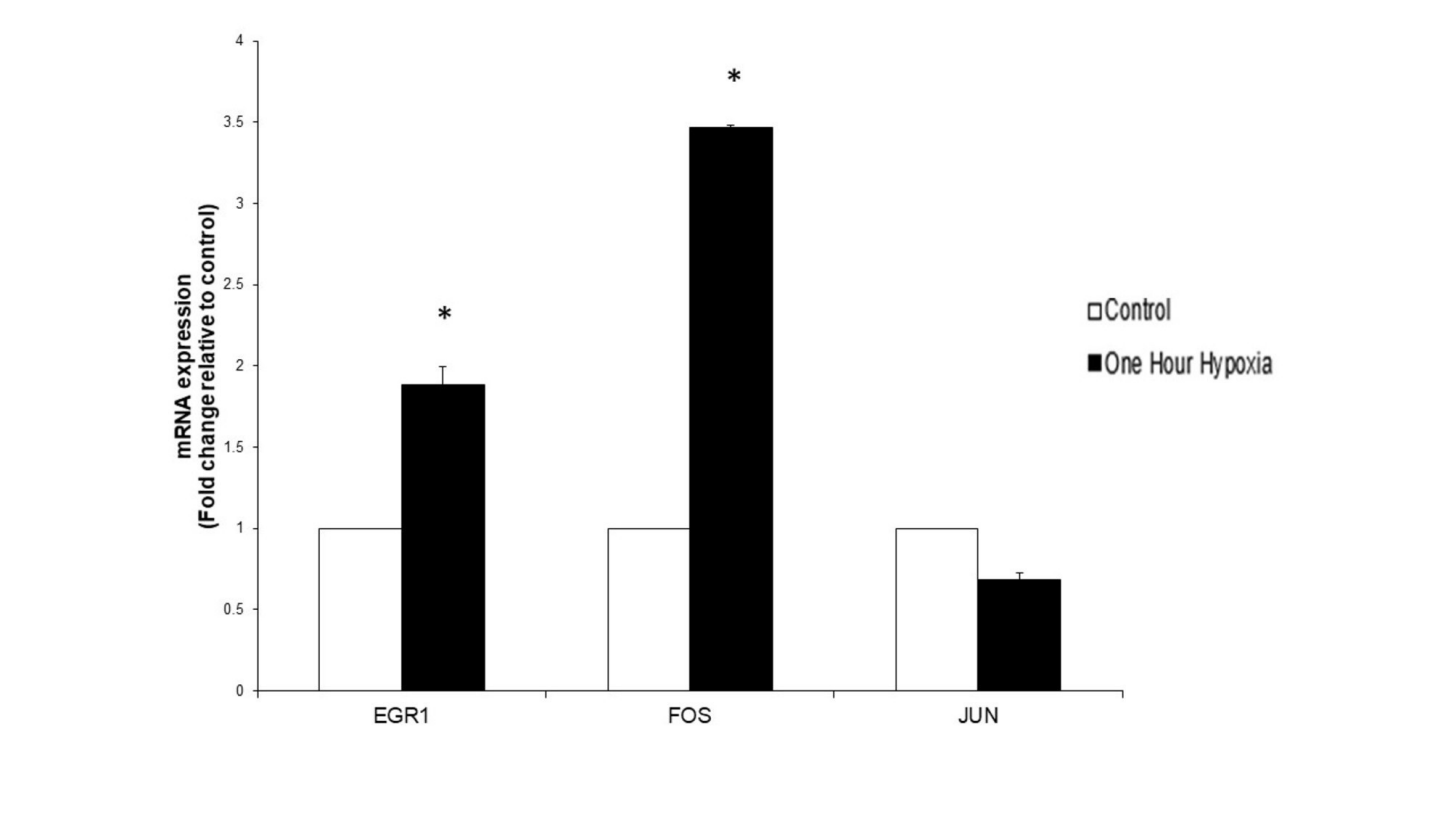
The effect of one hour of simulated ischaemia on early response gene mRNA expression in primary human skeletal muscle cells. Analysis of early growth response 1 gene (EGR1), CJUN and CFOS mRNA expression was performed using real-time PCR. Gene expression is presented as a fold-change +/- Standard Deviation relative to control and represents the results of two independent experiments each carried out with triplicate samples. Means were compared using two-sample t-test. (* p-value < 0.05 compared to control).

Analysis of gene expression following one hour of simulated ischaemia showed an upregulation of EGR-1 and CFOS genes while CJUN was downregulated. EGR-1 was upregulated at 1.885 (p = 0.001), CFOS at 3.47 (p = <0.000), and CJUN was downregulated at 0.68 (p = 0.197) (Figure 6). Our in vitro simulated ischaemia showed somewhat similar results to those on microarray data in that EGR-1 and CFOS were upregulated on both microarray data and following simulated ischaemia (Table 2).

**Table 2:** Genes upregulated on microarray at one hour due to ischaemia compared to one hour simulated ischaemia using hypoxic chamber. These values represent the fold-change relative to control samples. EGR-1 = Early growth response 1 gene

Gene	Microarray	Hypoxic Chamber
EGR1	3.64	1.89
CJUN	5.11	0.68
CFOS	55.55	3.47

Analysis of gene expression following two hours of simulated ischaemia showed a downregulation of EGR-1 at 0.73 (p = 0.5681). CFOS remained upregulated compared to controls at 1.46 (p = 0.832). Though persistently upregulated, the fold change of CFOS represents a decrease from the level of mRNA expression after one-hour ischaemia. Expression of CJUN was only minimally upregulated at 1.06 (p = 0.703) compared to controls.

**Figure 7:**
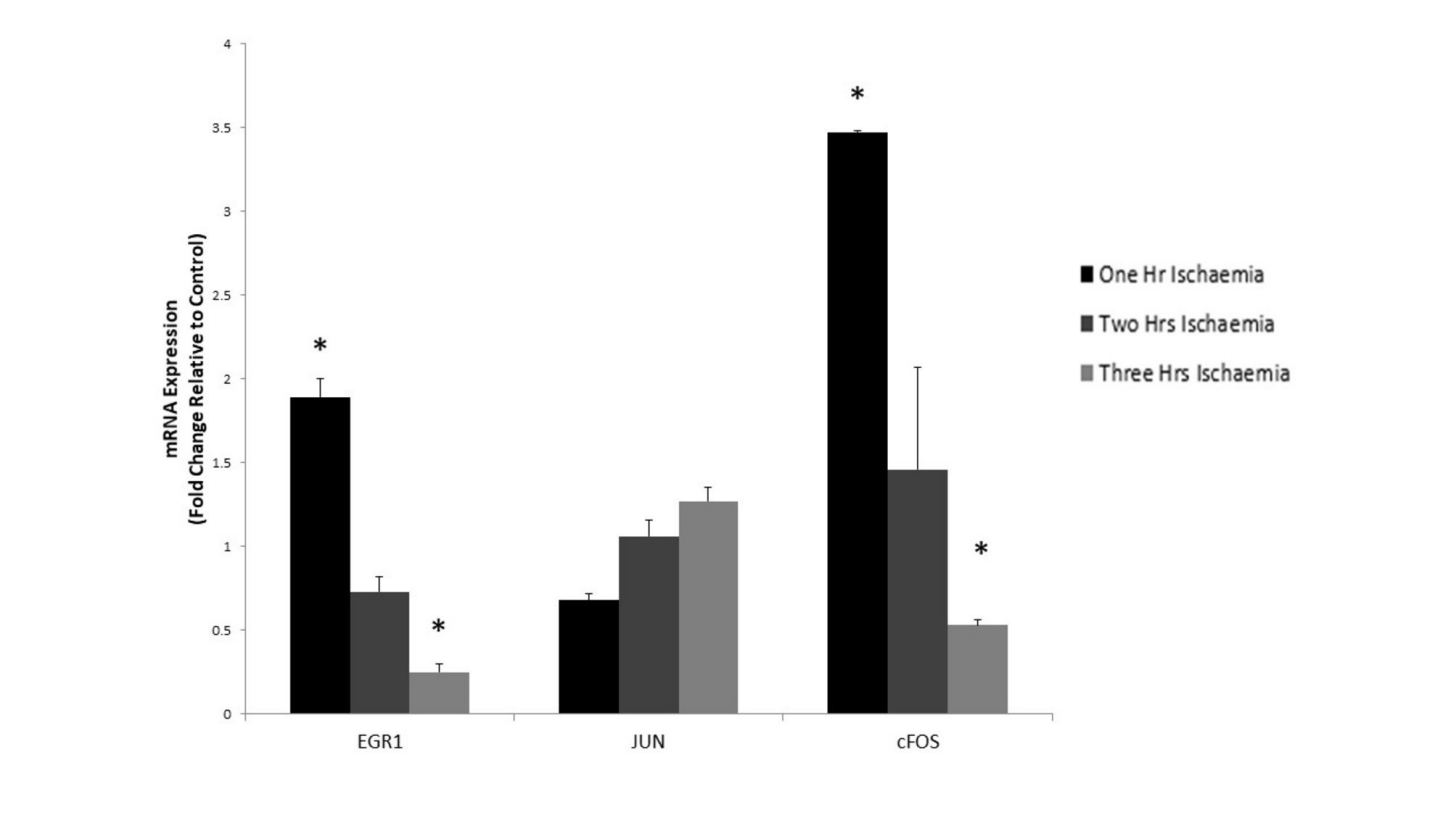
The effect of one, two and three hours of simulated ischaemia on the expression of early response genes in primary human skeletal muscle cells. Analysis of early growth response 1 gene (EGR1), CJUN and CFOS mRNA expression was performed using real-time PCR. Gene expression is presented as a fold-change +/- Standard Deviation relative to control and represents the results of two independent experiments each carried out with triplicate samples. Means were compared using two-sample t-test, Mann-Whitney U-test and Welch’s t-test. (* p-value < 0.05 compared to control)

Following three hours of simulated ischaemia, mRNA expression of EGR-1 and CFOS was downregulated compared to controls. The fold-changes of these genes were 0.25 (p < 0.000) and 0.53 (p < 0.000), respectively. CJUN was further upregulated at 1.27 (p = 0.6295) compared to controls. Therefore, the greatest fold change for EGR-1 and CFOS occurred following one hour of simulated ischaemia and then decreased with prolonged ischaemia. This behavior corresponds to that which is expected. CJUN is slightly unusual in that it continues to be expressed at greater levels with prolonged periods of simulated ischaemia (Figure 7).

IPC + Hypoxia

**Figure 8:**
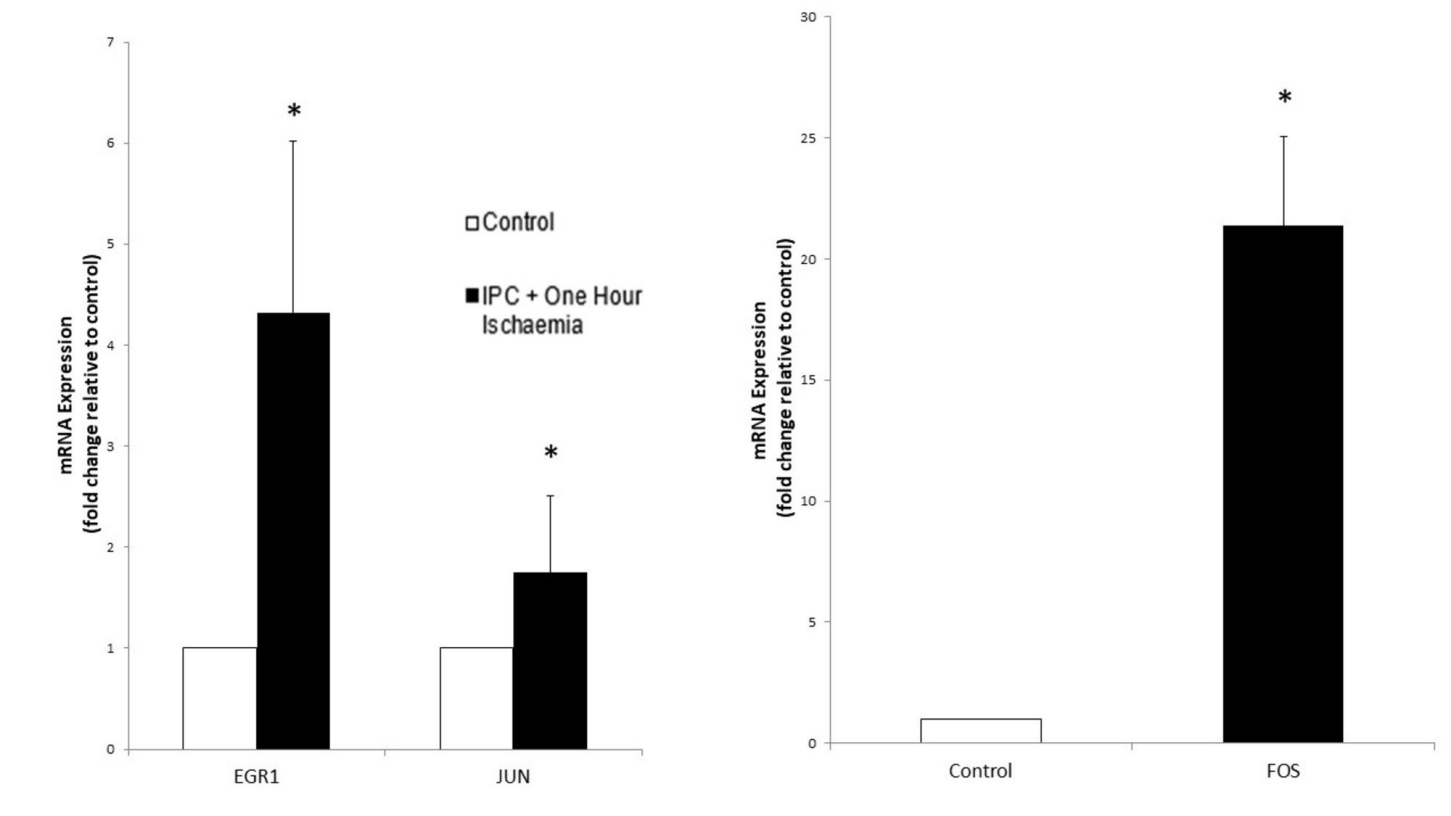
The effect of simulated ischaemia on the expression of early response gene mRNA in primary human skeletal muscle cells following ischaemic preconditioning (IPC) and one hour of ischaemia. Analysis of early growth response 1 gene (EGR1), CJUN and CFOS mRNA expression was performed using real-time PCR. Gene expression is presented as a fold-change +/- Standard Deviation relative to control and represents the results of two independent experiments each carried out with triplicate samples. Means were compared using two-sample t-test and Mann-Whitney U-test. (* p-value < 0.05 compared to control)

Analysis of gene expression following simulated IPC and subsequent prolonged ischaemia for one hour demonstrated upregulation of all genes. CFOS was upregulated to the greatest extent at a 21.37-fold change compared to controls (p < 0.000). EGR-1 was upregulated at 4.32 (p < 0.000) and CJUN at 1.75 (p = 0.047) (Figure 8). These results were again similar to microarray data (Table 3).

**Table 3:** Genes upregulated on microarray at one hour ischaemia following ischaemic preconditioning (IPC) compared to simulated IPC and one hour ischaemia using hypoxic chamber. These values represent the fold-change relative to control samples. EGR-1 = Early growth response 1 gene

Gene	Microarray	Hypoxic Chamber
EGR1	2.84	4.32
CJUN	1.41	1.75
CFOS	2.11	21.37

**Figure 9:**
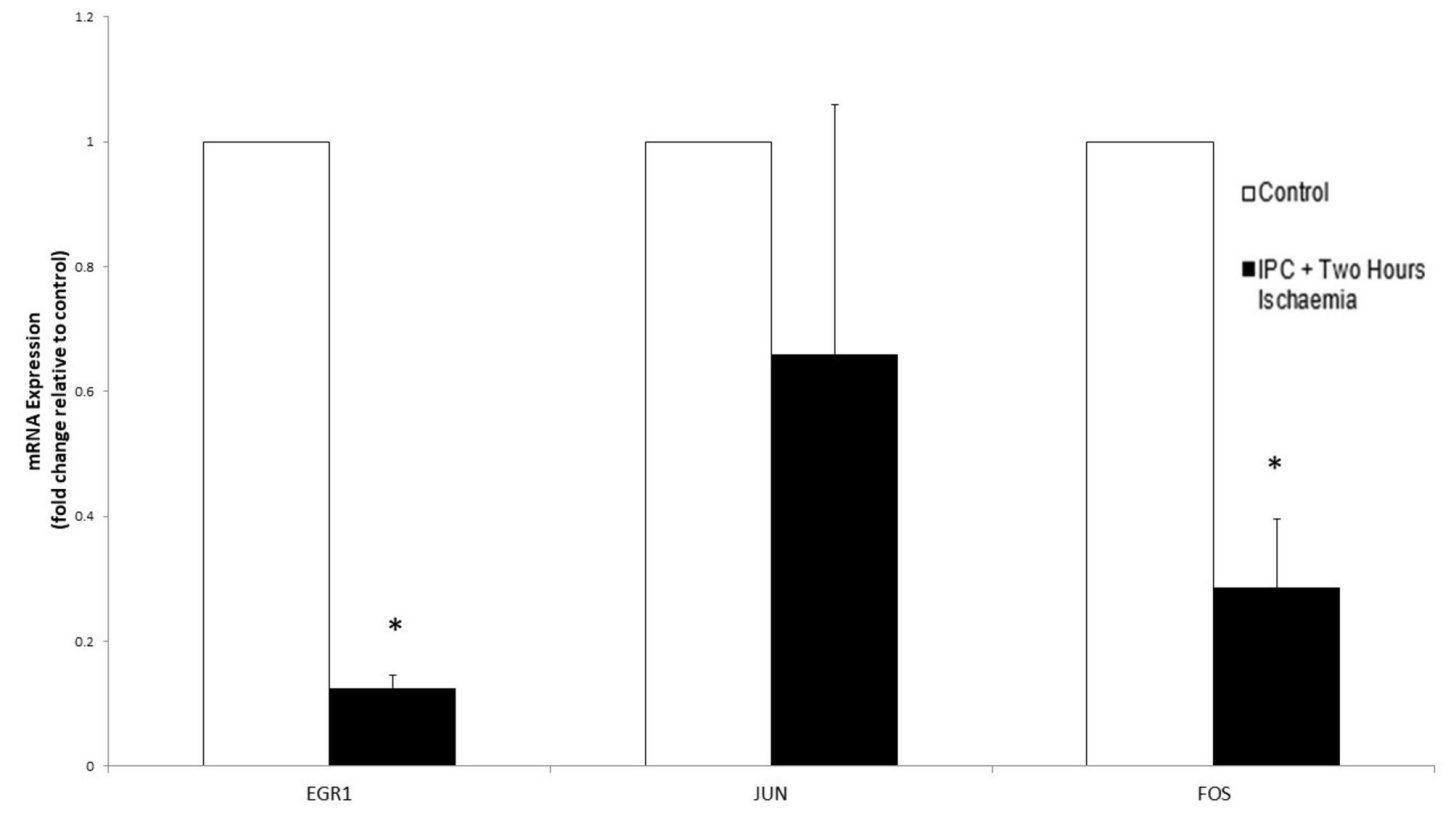
The effect of simulated ischaemia on the expression of early response gene mRNA in primary human skeletal muscle cells following ischaemic preconditioning (IPC) and two hours of ischaemia. Analysis of early growth response 1 gene (EGR1), CJUN and CFOS mRNA expression was performed using real-time PCR. Gene expression is presented as a fold-change +/- Standard Deviation relative to control and represents the results of two independent experiments each carried out with triplicate samples. Means were compared using two-sample t-test and Mann-Whitney U-test. (* p-value < 0.05 compared to control)

Gene expression analysis following simulated IPC and two hours of ischaemia revealed a downregulation of all three genes. EGR-1 was downregulated at a 0.125-fold change (p < 0.000) while the fold changes of CJUN and CFOS were 0.66 (p = 0.1112) and 0.285 (p = 0.0002), respectively (Figure 9 and Table 4).

**Table 4:** Comparison of early response gene expression following simulated IPC and ischaemia versus simulated ischaemia only. Gene expression is presented as a fold-change relative to control (EGR-1 = Early growth response 1 gene). Means were compared using two-sample t-test and Mann-Whitney U-test. P values relate to the difference between the means of the ischaemia only and the IPC + ischaemia mRNA expression (* p-value < 0.05). IPC: Ischaemic preconditioning

Duration of Simulated Ischaemia	Gene	Ischaemia Only	IPC + Ischaemia	p-value
One Hour	EGR1	1.885	4.32	<0.000*
	CJUN	0.68	1.75	0.01*
	CFOS	3.47	21.37	<0.000*
Two Hours	EGR-1	0.73	0.125	0.4063
	CJUN	1.06	0.66	0.0391*
	CFOS	1.46	0.285	0.004*

## Discussion

Use of hypoxic model

The establishment of a reproducible in vitro model is an integral step in investigating IPC in skeletal muscle. We used an air-tight resealable chamber connected to 1% oxygen and hypoxic media to produce a hypoxic environment (Figure 1). Our group has previously published on the use of this hypoxic chamber for the investigation of IPC in muscle cells [18].

By showing that HIF-1 α expression was upregulated in skeletal muscle cells following exposure to this simulated ischaemic environment, we confirm that our experimental conditions are indeed accurate at reproducing significant hypoxia. HIF-1 α should be upregulated early following exposure to hypoxic conditions but this upregulation should only be transient [24, 29]. Therefore, our results show that hypoxia is satisfactorily transiently induced following simulated ischaemia via the hypoxic chamber and buffer solution. This was the basis for our subsequent experiments.

Genomic response of skeletal muscle to IPC

In deciding relevant time points to assess gene expression following IPC, we focused on clinical relevance. In clinical practice, many surgical procedures carried out on the extremities are done so under tourniquet-control. However, it is advised that tourniquet time should be limited to two hours as risk of compression neurapraxia is greatly increased with greater than two hours tourniquet use. Therefore, gene expression analysis was carried out following simulated ischaemic preconditioning and one or two hours of hypoxia.

Early Response Genes

Expression of early response genes EGR-1, CJUN and CFOS was examined following simulated ischaemia of one, two and three-hour durations. Expression of EGR1 and CFOS was upregulated and peaked after one hour of hypoxia (p = 0.001 and p = <0.00, respectively). Expression of EGR-1 and CFOS mRNA then decreased with subsequent prolonged periods of ischaemia so that they were downregulated after three hours of simulated ischaemia. These results exhibit an appropriate response of these genes to ischaemia. Early response genes would be expected to be upregulated early but prolonged upregulation of proto-oncogene, such as CFOS, may be detrimental to cells and result in apoptosis. These results are also consistent with previous studies on early response genes [19, 26].

CJUN, as an early response gene, did not behave as expected. It was initially downregulated following one hour of simulated ischaemia. Expression of CJUN mRNA then increased steadily with both two and three hours of simulated ischaemia. The profile of CJUN expression is conflicting when compared to the other early response genes. Though this is unexpected, previous work has shown CJUN to act in a similar way. Mehiri et al. looked at the expression of several genes following diaphragmatic injury in a rat model including CJUN [30]. In this study, CJUN mRNA expression decreased initially post-injury, returned to baseline after 12 hours and peaked at 96 hours post-injury. Though CJUN is a known transcription factor and thought of as an early response gene, it may not behave in the same way as other early response genes.

Overall, our results show expected gene expression profiles following simulated ischaemia. These results are similar to microarray data [19]. Therefore, we are satisfied that our experimental methodology was sound, and the genes reviewed were appropriate.

IPC prior to simulated ischaemia, produced a greater level of upregulation of EGR1, JUN and FOS genes (p = <0.00, 0.047, and <0.00, respectively) at one hour compared to simulated ischaemia alone. These genes were then all downregulated after simulated IPC and two hours of ischaemia. Simulated IPC produced a greater upregulation of these genes initially and a more rapid decline in their expression with prolonged ischaemia. In summary, IPC confers greater protection to skeletal muscle cells as it results in a greater early expression of protective genes but also an earlier downregulation of these genes thus preventing the complications of prolonged expression of these genes (Table 4).

Clinical potential

Ischaemic preconditioning has significant clinical potential. It has been shown to protect against subsequent prolonged ischaemia and ischaemia-reperfusion injury both in animal and human models [1, 4, 9-12]. This is very relevant in orthopaedics where many elective and trauma procedures are performed under tourniquet control. IPC may have the benefit of enabling a surgeon to operate under a slightly longer tourniquet time than the current standard of two hours by reducing the ischaemic insult to a limb from prolonged tourniquet time. This may mean that more complex operations could be performed under the safety of tourniquet control. However, tourniquet time would still be limited as IPC does not protect cells indefinitely. Even in Murry’s landmark initial study on IPC [1], canine hearts were protected by IPC when subjected to 40 mins of sustained ischaemia but when the myocardium was rendered ischaemic for three hours, the protective effect was lost.

If the protective effect of IPC could be induced for an extended period of time without exposing cells to repetitive ischaemia-reperfusion, the clinical benefits could be colossal. For instance, direct activation of the cellular pathways involved in IPC by therapeutic drugs would enable skeletal muscle protection without the necessity of undergoing an ischaemic preconditioning insult. A clear understanding of the mechanisms involved in IPC is essential to allow for methodical pharmacological development.

## Conclusions

Our findings lend support to the use of our hypoxic chamber and protocol of ischaemic preconditioning for future in vitro studies on skeletal muscle preconditioning. It is another device in our armamentarium in understanding and clinically applying the complex protective mechanism of IPC. We have demonstrated that IPC induces a protective genomic response. Cells exposed to IPC prior to periods of ischaemia show upregulation of protective genes when compared to control samples. EGR1, CJUN and CFOS have all been shown to protect cells from ischaemic insult and are upregulated following IPC in our study. We would hope that future research will demonstrate meaningful benefits of IPC in the orthopaedic setting and improve clinical outcomes for patients.
